# Extraction of azimuthal asymmetries using optimal observables

**DOI:** 10.1140/epjc/s10052-019-6580-3

**Published:** 2019-01-21

**Authors:** Jörg Pretz, Fabian Müller

**Affiliations:** 1 Institut für Kernphysik, Forschungszentrum Jülich, 52425 Jülich, Germany; 2 III. Physikalisches Institut B, RWTH Aachen University, 52056 Aachen, Germany; 3 JARA-FAME, Forschungszentrum Jülich und RWTH Aachen University, Jülich, Germany

## Abstract

Azimuthal asymmetries play an important role in scattering processes with polarized particles. This paper introduces a new procedure using event weighting to extract these asymmetries. It is shown that the resulting estimator has several advantages in terms of statistical accuracy, bias, assumptions on acceptance and luminosities compared to other estimators discussed in the literature.

## Introduction and motivation

This paper describes the extraction of an azimuthal asymmetry $\epsilon$ occurring in an event distribution given by

1 $$\begin{aligned} N(\vartheta ,\varphi ) = \frac{1}{2\pi } \, {\mathcal {L}} \, a(\vartheta ,\varphi ) \, \sigma _0(\vartheta ) \, (1 + \epsilon (\vartheta ) \cos (\varphi )) . \end{aligned}$$

The variables in Eq. (1) are defined in Table 1. Event distributions of this type appear for example in scattering processes of a transversally polarised beam on a spin 0 target [1]. The parameter $\epsilon$ is the product of the polarisation and an analyzing power, $\epsilon = P A$. Once $\epsilon$ is determined one can either determine the polarisation *P* if the analyzing power *A* is known, or vice versa. To cancel systematic effects, one usually takes two data sets with opposite polarisations, e.g. polarisation up ($P^\uparrow$) and down ($P^\downarrow$). The acceptance factor $a(\vartheta , \varphi )$ may have an arbitrary dependence on the $\varphi$ and $\vartheta$. The only assumption is that the acceptance is the same for the two data sets.

In this paper a new estimator using event weights and a $\chi ^2$-minimization is introduced. The method is an application of optimal observables discussed in Refs. [2, 3], but it also takes into account luminosity and acceptance effects. The paper is organized as follows. In Sect. 2 several estimators to determine $\epsilon$ (i.e. *P* or *A*) are discussed and compared. Section 2.2 introduces the new method. Possible extensions of this new weighting/fitting method are discussed in Sect. 3.

## Estimators to determine azimuthal asymmetries

In general one can distinguish two classes of estimators: estimators using event counts, discussed in Sect. 2.1 and estimators using event weights, discussed in Sect. 2.2.

### Estimators using event counts

**Fig. 1:**
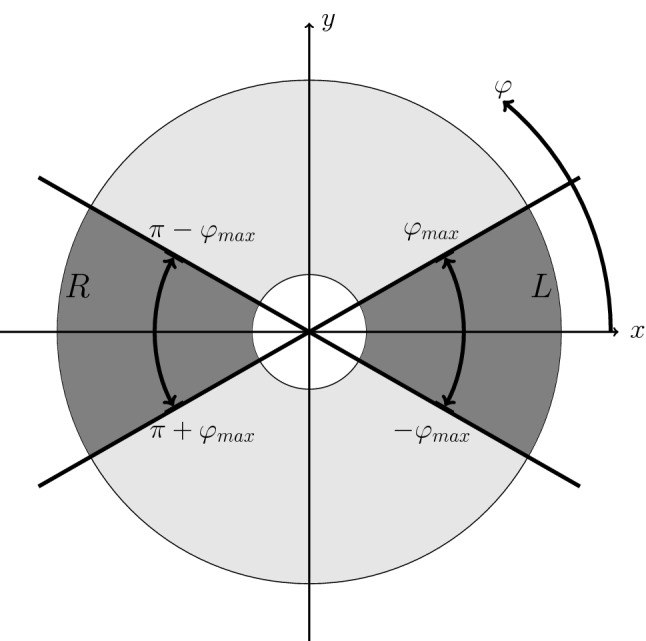
Definition of azimuthal angle and accepted events. The beam moves in *z*-direction, i.e. out of the plane

Here events around $\varphi =0$ and $\varphi =\pi$ as indicated by the dark region in Fig. 1 enter the analysis. The expectation value for the number of events in the left (*L*) part of the detector is given by:

2 $$\begin{aligned} \left\langle N_L^\uparrow \right\rangle= & {} \frac{1}{2\pi } \, \int _{-\varphi _{max}}^{\varphi _{max}} {\mathcal {L}}^{\uparrow } a(\varphi ) \sigma _0 \left( 1 + P^\uparrow A \cos (\varphi ) \right) \mathrm {d}\varphi \end{aligned}$$

3 $$\begin{aligned}= & {} {\mathcal {L}}^{\uparrow } a_L \sigma _0 \left( 1 + \left\langle \cos (\varphi ) \right\rangle _L P^\uparrow A \right) \end{aligned}$$

with

$$\begin{aligned} a_L= & {} \frac{1}{2\pi } \, \int _{-\varphi _{max}}^{\varphi _{max}} a(\varphi ) \mathrm {d}\varphi \quad \text{ and } \\ \left\langle \cos (\varphi ) \right\rangle _L= & {} \frac{\int _{-\varphi _{max}}^{\varphi _{max}} a(\varphi ) \cos (\varphi ) \mathrm {d}\varphi }{\int _{-\varphi _{max}}^{\varphi _{max}} a(\varphi ) \mathrm {d}\varphi } . \end{aligned}$$

To simplify the notation the $\vartheta$-dependence is dropped. Similar equations exist for $\left\langle N_R^\uparrow \right\rangle$, $\left\langle N_L^\downarrow \right\rangle$ and $\left\langle N_R^\downarrow \right\rangle$.

**Table 1:** Definitions of variables used in Eq. (1)

Variable	Meaning
$N(\vartheta ,\varphi )$	Number of events observed
$\left\langle N(\vartheta ,\varphi ) \right\rangle$	Expectation value of number of events
$\sigma _0(\vartheta )$	Unpolarized cross section
$\vartheta$	Polar angle
$\varphi$	Azimuthal angle, $\varphi =0$ corresponds to positive *x*-direction
$\epsilon = PA$	Asymmetry parameter to be determined
*P*	Beam polarization
$A(\vartheta )$	Analyzing power
${\mathcal {L}}$	Luminosity
$a(\vartheta ,\varphi )$	Acceptance

In the cross ratio

4 $$\begin{aligned} \delta= & {} \frac{\left\langle N^{\uparrow }_L \right\rangle \left\langle N^{\downarrow }_R \right\rangle }{\left\langle N^{\uparrow }_R \right\rangle \left\langle N^{\downarrow }_L \right\rangle } \nonumber \\= & {} \frac{(1 + \left\langle \cos (\varphi ) \right\rangle _L P^{\uparrow } A) (1 + \left\langle \cos (\varphi ) \right\rangle _R P^{\downarrow } A)}{(1 + \left\langle \cos (\varphi ) \right\rangle _R P^{\uparrow } A) (1 + \left\langle \cos (\varphi ) \right\rangle _L P^{\downarrow } A)} \, \end{aligned}$$

introduced in Ref. [4], the usually unknown luminosities, acceptances and unpolarized cross section cancel. Replacing the expectation values by the actual measured event counts, the following estimator for the analyzing power *A* can be derived

5 $$\begin{aligned} {\hat{A}}= & {} \frac{X-\sqrt{X^2- 2 Y (\delta -1)}}{Y} , \quad \text{ with } \\ X= & {} P^\downarrow \left( \left\langle \cos (\varphi ) \right\rangle _R - \left\langle \cos (\varphi ) \right\rangle _L \delta \right) \nonumber \\&+ P^\uparrow \left( \left\langle \cos (\varphi ) \right\rangle _L - \left\langle \cos (\varphi ) \right\rangle _R \delta \right) \quad \text{ and } \nonumber \\ Y= & {} 2 \left\langle \cos (\varphi ) \right\rangle _L \left\langle \cos (\varphi ) \right\rangle _R P^\downarrow P^\uparrow (\delta -1).\nonumber \end{aligned}$$

Note that to evaluate $\left\langle \cos (\varphi ) \right\rangle _{L,R}$ information on the acceptance is needed. This method was for example applied in Ref. [5]. Here bins of $\varDelta \varphi =\pm \, 30^{\circ }$ were used.

Another possibility is to consider estimators of the type

6 $$\begin{aligned} {\hat{A}}= & {} \frac{1}{P \left\langle \cos (\varphi ) \right\rangle } \, \frac{N_L^{\uparrow (\downarrow )}- N_R^{\uparrow (\downarrow )}}{N_L^{\uparrow (\downarrow )} + N_R^{\uparrow (\downarrow )}} \quad \quad \text{ or }\nonumber \\ {\hat{A}}= & {} \frac{1}{P \left\langle \cos (\varphi ) \right\rangle } \, \frac{N_{L(R)}^\uparrow - N_{L(R)}^\downarrow }{N_{L(R)}^\uparrow + N_{R(L)}^\downarrow } \end{aligned}$$

where various corrections have to be applied in order to compensate for acceptance and luminosity difference between the two data sets. These type of estimators were used in Refs. [6, 7].

Common to these estimators is that they reach the same statistical error $\sigma$. In general it is more convenient to work with the figure of merit (FOM) defined by $\text{ FOM } = \sigma ^{-2}$. To evaluate the FOM we make a few assumptions to simplify the notation: First, $P^\uparrow = -P^\downarrow$, in addition we assume that one takes roughly the same number of events in both polarisation configurations. We also assume a uniform acceptance in $\varphi$. It is straight forward to derive formulas dropping these assumptions but the expressions are getting cumbersome. These assumptions do not change the overall conclusions comparing different estimators. Instead of discussing the FOM on *A*, we will discuss the FOM of $\epsilon$.

Error propagation from Eqs. (5) or (6) leads to

7 $$\begin{aligned} \text{ FOM }_{\epsilon }^{\mathrm {counts}} = N_{\mathrm {tot}} \, \frac{\left\langle \cos (\varphi ) \right\rangle ^2}{1-\left\langle \cos (\varphi ) \right\rangle ^2 \epsilon ^2} \, \end{aligned}$$

where $N_{\mathrm {tot}}$ is the total number of events entering the analysis. Details of the calculation are given in Appendix B.1. Neglecting the term with $\epsilon$, one finds:

8 $$\begin{aligned} \text{ FOM }^{\mathrm {counts}}_\epsilon= & {} N_{\mathrm {tot}} \, \left\langle \cos (\varphi ) \right\rangle ^2 \end{aligned}$$

9 $$\begin{aligned}= & {} N_0 \, \frac{2 \varphi _{max}}{ \pi } \, \left( \frac{ \int _{-\varphi _{max}}^{\varphi _{max}} \cos (\varphi )\mathrm {d}\varphi }{ \int _{-\varphi _{max}}^{\varphi _{max}} \mathrm {d}\varphi } \right) ^2 \nonumber \\= & {} N_0 \, \frac{2 \sin ^2(\varphi _{max})}{\pi \varphi _{max}} \end{aligned}$$

where $N_0 = \int _0^{2 \pi } a \sigma _0 ({\mathcal {L}}^\uparrow + {\mathcal {L}}^\downarrow ) \mathrm {d}\varphi$ is the total number of events available in both polarisation states. Thus $N_{\mathrm {tot}} = N_0 (2 \varphi _{max})/(\pi )$ is the total number of events entering the analysis.

**Fig. 2:**
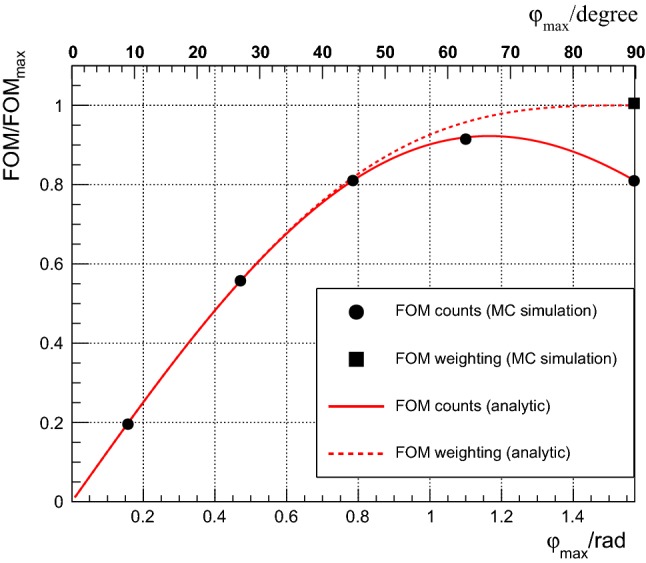
Figure of merit (FOM) for estimators using event counts and event weighting, calculated analytically (lines) and from MC simulation (symbols)

The full line in Fig. 2 shows the FOM calculated according to Eq. (9) for different $\varphi$-ranges. Increasing $\varphi _{max}$, the FOM increases first. Around $\varphi _{max}\approx 65^{\circ }$ it starts to decrease. The reason is that one adds more and more events where $\cos (\varphi )$ is small. These events carry less information on $\epsilon$ and dilute the sample in the way the analysis is performed. This clearly shows that this cannot be the optimal strategy. In the next section estimators will be discussed where the FOM reaches the dashed line, which corresponds to the Cramér–Rao bound.

### Estimators using event weights

In this section estimators are discussed which use event weights instead of event counts as in the previous subsection. In Ref. [8] weighted sums $\sum _i \cos (\varphi _i)$ are introduced in order to extract $\epsilon$. To cancel acceptance effects the authors propose to combine the event distributions from the two polarisation states. They do not address the question how to deal with different luminosities in the two different polarisation states. The method was applied in Ref. [9] where an azimuthal symmetry of the detector is assumed. It is also shown in Ref. [8] that with this weighting procedure the FOM reaches the Cramér–Rao bound as does the unbinned likelihood method. An unbinned likelihood method was used in Ref. [10]. It is not straight forward to apply because the probability density function is not completely known. Acceptance effects have to be verified using a Monte Carlo simulation.

Now a new method, reaching the Cramér–Rao bound as well, is introduced. The advantage is that no knowledge about the acceptance is required (as long as it is the same for both data sets, as in any other method) and no corrections concerning the luminosities have to be applied. On the contrary, information on the acceptance and luminosity factor ${\mathcal {L}} \sigma _0 a_0$ are obtained in parallel to $\epsilon$ in this method.

We consider the following six observables

$$\begin{aligned} \sum _{i=1}^{N^\uparrow } \cos ^n(\varphi _i) \quad \text{ and } \quad \sum _{i=1}^{N^\downarrow } \cos ^n(\varphi _i) , \text{ with } \quad n=0,1,2 . \end{aligned}$$

The sums run over the number of events in the given polarisation state including all azimuthal angels. Note that $n=0$ corresponds just to the number of events observed, $n=1 (2)$ are higher moments and correspond to the sum over events weighted with $\cos (\varphi ) (\cos ^2(\varphi ))$.

For an arbitrary acceptance in $\varphi$ we can write the following Fourier series:

10 $$\begin{aligned} a(\varphi ) = a_0 + \sum _{n=1}^{\infty } a_n \cos (n \varphi ) + b_n \sin (n \varphi ) . \end{aligned}$$

The expectation values of these observables are given by

11 $$\begin{aligned} \left\langle N^\uparrow \right\rangle= & {} \frac{1}{2\pi } \, \mathcal {L^\uparrow }\sigma _0 \int _0^{2 \pi } \left[ a_0 + \sum _{i=n}^{\infty } a_n \cos (n \varphi ) + b_n \sin (n \varphi ) \right] \nonumber \\&\times \left( 1 + P^\uparrow A \cos (\varphi ) \right) \mathrm {d}\varphi \nonumber \\= & {} \mathcal {L^\uparrow } \sigma _0 a_0 \left( 1 + \frac{a_1}{2 a_0} P^\uparrow A \right) , \end{aligned}$$

12 $$\begin{aligned}&{\left\langle \sum _\uparrow \cos (\varphi _i) \right\rangle } = \frac{1}{2\pi } \, \mathcal {L^\uparrow } \sigma _0\int _0^{2 \pi } \cos (\varphi ) \nonumber \\&\qquad \times \left[ a_0 + \sum _{n=1}^{\infty } a_n \cos (n \varphi ) + b_n \sin (n \varphi ) \right] \nonumber \\&\qquad \times \left( 1 + P^\uparrow A \cos (\varphi _i) \right) \mathrm {d}\varphi \nonumber \\&\quad = \frac{1}{2} \, {\mathcal {L}}^\uparrow \sigma _0 a_0 \left( P^\uparrow A \left( 1 + \frac{a_2}{2 a_0} \right) + \frac{a_1}{a_0} \right) , \end{aligned}$$

13 $$\begin{aligned}&{\left\langle \sum _\uparrow \cos ^2(\varphi _i) \right\rangle } = \frac{1}{2\pi } \, \mathcal {L^\uparrow } \sigma _0 \int _0^{2 \pi } \cos ^2(\varphi ) \nonumber \\&\qquad \times \left[ a_0 + \sum _{n=1}^{\infty } a_n \cos (n \varphi ) + b_n \sin (n \varphi ) \right] \nonumber \\&\qquad \times \left( 1 + P^\uparrow A \cos (\varphi ) \right) \mathrm {d}\varphi \nonumber \\&\quad = \frac{1}{2} \, \mathcal {L^\uparrow } \, \sigma _0 a_0 \left( \left( 1+ \frac{a_2}{2a_0} \right) + \frac{1}{4} \, \frac{3 a_1+a_3}{a_0} P^\uparrow A \right) . \end{aligned}$$

Similar expressions hold for the expectation values $\left\langle N^\downarrow \right\rangle , \left\langle \sum _\downarrow \cos (\varphi _i) \right\rangle , \left\langle \sum _\downarrow \cos ^2(\varphi _i) \right\rangle$ of the second polarisation state by replacing $P^\uparrow$ with $P^\downarrow$. The integrals extend over all azimuthal angles from 0 to 2$\pi$. It is also possible to apply the method for a limited range as in the previous section. In this case the integrals would extend over $[-\varphi _{max},\;\varphi _{max}]$ and $[\pi -\,\varphi _{max},\;\pi \,+\,\varphi _{max}]$ (dark region in Fig. 1).

Assuming that the polarisations $P^\uparrow$ and $P^\downarrow$ are known, using a $\chi ^2$ minimization comparing the expectation values with the observables, one can determine the following 6 unknown parameters:

$$\begin{aligned} ({\mathcal {L}}^\uparrow \sigma _0 a_0), ({\mathcal {L}}^\downarrow \sigma _0 a_0), \frac{a_1}{a_0}, \frac{a_2}{a_0}, \frac{a_3}{a_0}, A . \end{aligned}$$

The $\chi ^2$ is given by:

14 $$\begin{aligned} \chi ^2= & {} (\mathbf {y}_{\mathrm {obs}} - \mathbf {y}_{\mathrm {model}}) \, C^{-1} \, (\mathbf {y}_{\mathrm {obs}} - \mathbf {y}_{\mathrm {model}})^T \end{aligned}$$

with

$$\begin{aligned} \mathbf {y}_{\mathrm {obs}}= & {} \left( N^\uparrow , \sum _{\uparrow } \cos (\varphi _i),\sum _{\uparrow } \cos ^2(\varphi _i), \right. \nonumber \\&\left. N^\downarrow ,\sum _{\downarrow } \cos (\varphi _i),\sum _{\downarrow } \cos ^2(\varphi _i) \right) , \\ \mathbf {y}_{\mathrm {model}}= & {} \left( {\left\langle N^\uparrow \right\rangle },{\left\langle \sum _{\uparrow } \cos (\varphi _i) \right\rangle },{\left\langle \sum _\uparrow \cos ^2(\varphi _i) \right\rangle }, \right. \nonumber \\&\left. {\left\langle N^\downarrow \right\rangle }, {\left\langle \sum _\downarrow \cos (\varphi _i) \right\rangle },{\left\langle \sum _\downarrow \cos ^2(\varphi _i) \right\rangle } \right) . \end{aligned}$$

The covariance matrix *C* of the observables is given in Appendix A. The easiest way to obtain values for the parameters is to minimize Eq. (14) numerically although analytic, but cumbersome, expressions exist for the parameters. The numerical solution is also preferred in view of possible extensions of the method discussed in Sect. 3, where analytic solutions may not exist.

The FOM, calculated using the same conditions as used for $\text{ FOM }^{\mathrm {counts}}_\epsilon$ in Eq. (9), is derived in Appendix B. The final result is:

15 $$\begin{aligned} \text{ FOM }^{\mathrm {weighting}}_\epsilon = N_{\mathrm {tot}} \frac{\left\langle \cos ^2(\varphi ) \right\rangle ^2}{\left\langle \cos ^2(\varphi ) \right\rangle - \left\langle \cos ^4(\varphi ) \right\rangle \epsilon ^2} . \end{aligned}$$

Neglecting the term with $\epsilon$ one finds:

16 $$\begin{aligned}&\text{ FOM }^{\mathrm {weighting}}_\epsilon = N_{\mathrm {tot}} \left\langle \cos ^2{\varphi } \right\rangle \end{aligned}$$

17 $$\begin{aligned}&= N_0 \, \frac{ 2 \varphi _{max}}{\pi } \, \frac{\int _{-\varphi _{max}}^{\varphi _{max}} \cos ^2(\varphi ) \mathrm {d}\varphi }{ \int _{-\varphi _{max}}^{\varphi _{max}} \mathrm {d}\varphi } \nonumber \\&= N_0 \, \frac{\varphi _{max} + \sin (\varphi _{max}) \cos (\varphi _{max})}{ \pi } . \end{aligned}$$

It is shown as a dashed line in Fig. 2. At small $\varphi _{max}$ the FOM of counting and weighting estimators coincide, at larger $\varphi _{max}$, $\text{ FOM }^{\mathrm {weighting}}_\epsilon$ keeps increasing.

### General discussion on the figure of merit

In this subsection we make some general remarks about the FOM reachable for event distributions of the type

18 $$\begin{aligned} n(\varphi ) = \alpha (\varphi ) \left( 1 \pm \beta (\varphi ) \epsilon \right) . \end{aligned}$$

As shown in Ref. [11] the estimator

19 $$\begin{aligned} {\hat{\epsilon }} = \frac{\sum _\uparrow w(\varphi _i) - \sum _\downarrow w(\varphi _i)}{\sum _\uparrow w(\varphi _i) \beta (\varphi _i) + \sum _\downarrow w(\varphi _i) \beta (\varphi _i)} \end{aligned}$$

is bias free, where $w(\varphi )$ is an arbitrary weight function. The FOM is given by

$$\begin{aligned} \text{ FOM }^{w}_{\epsilon } = N_{\mathrm {tot}} \frac{\left\langle w \beta \right\rangle ^2}{\left\langle w^2(1-\epsilon ^2 \beta ^2) \right\rangle } . \end{aligned}$$

The choice $w=1$, or to be more precise $w=1$ if the event enters the analysis and $w=0$ else, results in

20 $$\begin{aligned} \text{ FOM }_{\epsilon }^{w=1} = N_{\mathrm {tot}} \frac{\left\langle \beta \right\rangle ^2}{\left\langle (1-\epsilon ^2 \beta ^2) \right\rangle } . \end{aligned}$$

The choice $w=\beta$ leads to the largest FOM (in the limit $\epsilon \ll 1$) reaching the Cramér–Rao bound:

21 $$\begin{aligned} \text{ FOM }_{\epsilon }^{w=\beta } = N_{\mathrm {tot}} \frac{\left\langle \beta ^2 \right\rangle ^2}{\left\langle \beta ^2(1-\epsilon ^2 \beta ^2) \right\rangle } . \end{aligned}$$

Translated to azimuthal asymmetries the factor $\beta (\varphi )$ equals $\cos (\varphi )$. The two FOMs given in Eq. (7), Sect. 2.1 and Eq.  (15), Sect. 2.2 are identical to the FOMs of Eqs. (20) and (21), respectively.

**Table 2:** Results of simulations

Parameter	Input value	Cross ratio, counting	Weighting/fit
Uniform acceptance			
*A*	0.2	$0.2030 \pm 0.0029$	$0.2030 \pm 0.0028$
$a_1/a_0$	0		$-\,0.0002 \pm 0.0014$
$a_2/a_0$	0		$-\,0.0002 \pm 0.0014$
$a_3/a_0$	0		$-\,0.0002 \pm 0.0028$
Non-uniform acceptance, Eq. (22)			
*A*	0.2	$0.1910 \pm 0.0031$	$0.2036 \pm 0.0031$
$a_1/a_0$	0.3		$0.3003 \pm 0.0013$
$a_2/a_0$	$-$ 0.3		$-\,0.3017 \pm 0.0013$
$a_3/a_0$	0.2		$0.2069 \pm 0.0025$

### Results of simulations

In this subsection we crosscheck the results of the previous subsections and discuss possible bias with the help of Monte Carlo simulations. A Monte Carlo simulation with $10^6$ events in total was performed by generating data according to Eq. (1) for two polarizations states with $P^\uparrow =0.5$ and $P^\downarrow =-\,0.5$ and $A=0.2$. The acceptance was once assumed to be uniform in $\varphi$ and once the following parameterization

22 $$\begin{aligned} a(\varphi )= & {} 1 + 0.3 \cos (\varphi ) \, \,-0.2 \sin (\varphi ) \nonumber \\&-\, 0.3 \cos (2\varphi ) + 0.1 \sin (2\varphi ) \nonumber \\&+\, 0.2 \cos (3\varphi ) + 0.2 \sin (3\varphi ) \nonumber \\&- \, 0.1 \cos (4\varphi ) + 0.1 \sin (4\varphi ) \, \end{aligned}$$

was used. In the analysis it is assumed that $a(\varphi )$ is unknown. Table 2 summarizes the results found using a MINUIT minimization in ROOT [12] to minimize $\chi ^2$ in Eq. (14). One sees that with the weighting/fitting method, one recovers the input analyzing power and the acceptance factors. No bias is observed. The cross ratio method, using events in the range $-1.2< \varphi _{max} <1.2$ to maximize the FOM (see Fig. 2), gives an unbiased result for *A* only in the case of uniform $\varphi$ acceptance as expected, since $\left\langle \cos (\varphi ) \right\rangle$ was calculated under this assumption.

The circles in Fig. 2 show the FOM obtained from the RMS of 1000 simulations where the analyzing power was calculated according to Eq. (5) for various values of $\varphi _{max}$. The square symbol is the FOM obtained from MINUIT using the weighting/fitting procedure. There is perfect agreement between the simulations and analytic formulas.

## Possible extensions

This subsection discusses some extensions which can be applied to the weighting/fitting method but in general not easily to the other methods.

If the polarisation vector points for example in an arbitrary unknown direction $\mathbf {P}=P(\cos (\varphi ),\sin (\varphi ))$ in the *x*-*y* plane, the observed signal is

23 $$\begin{aligned} N(\varphi )\propto & {} (1 + \epsilon _c \cos (\varphi ) + \epsilon _s \sin (\varphi )) . \end{aligned}$$

In this case, in the analysis one has to include also the sums

$$\begin{aligned} \sum _{\uparrow } \sin (\varphi _i)^n \quad \text{ and } \quad \sum _{\downarrow } \sin (\varphi _i)^n \quad \text{ for }\quad n=1,2 . \end{aligned}$$

This gives in total 10 equations for 10 unknowns. The unknowns are

$$\begin{aligned} (L^\uparrow \sigma _0 a_0), (L^\downarrow \sigma _0 a_0), \frac{a_1}{a_0}, \frac{a_2}{a_0}, \frac{a_3}{a_0}, \frac{b_1}{a_0}, \frac{b_2}{a_0}, \frac{b_3}{a_0}, \epsilon _c \, \, \text{ and } \, \, \epsilon _s . \end{aligned}$$

Including also tensor polarisation for a spin 1 particle, the event distributions reads

$$\begin{aligned} N(\varphi )\propto & {} (1 + \epsilon _c \cos (\varphi ) + \epsilon _s \sin (\varphi ) \\&+ \epsilon _{2c} \cos (2\varphi ) + \epsilon _{2s} \sin (2\varphi )) . \end{aligned}$$

This problem can be solved by using the observables

$$\begin{aligned} N,\, \sum _i \sin ^n(\varphi _i) , \, \sum _i \cos ^n(\varphi _i) , \quad \text{ for }\quad n=1,2,3,4 . \end{aligned}$$

for now in total three polarisation states. The number of equations increases to 27 for 19 parameters

$$\begin{aligned}&(L^\uparrow \sigma _0 a_0), (L^\downarrow \sigma _0 a_0), (L^0 \sigma _0 a_0),&\\&\frac{a_1}{a_0}, \frac{a_2}{a_0}, \frac{a_3}{a_0}, \frac{a_4}{a_0}, \frac{a_5}{a_0}, \frac{a_6}{a_0},&\\&\frac{b_1}{a_0}, \frac{b_2}{a_0}, \frac{b_3}{a_0}, \frac{b_4}{a_0}, \frac{b_5}{a_0}, \frac{b_6}{a_0},&\\&\epsilon _c , \epsilon _s , \epsilon _{2c} \,\, \text{ and } \, \, \epsilon _{2s} .&\end{aligned}$$

Looking at Eqs. (11)–(13), one observes that the parameter $a_3$ appears only once and even suppressed with respect to $a_1$ by a factor 3. One could set $a_3$ to zero resulting in a fit with 6 equations for 5 unknowns, which makes a $\chi ^2$ test possible. It is also possible to add a data set with unpolarized beam to the fit. This is for example useful if the two polarisations $P^\uparrow$ and $P^\downarrow$ are different and not known.

It is interesting to note that the method introduced here, especially for the case were the number of equations exceeds the number of parameter is a special case of the “Generalized Method of Moments” (GMM) widely used in economics (e.g. see Refs. [13, 14]).

## Summary and conclusion

Two types of estimators to extract azimuthal asymmetries have been compared. One is based on event counts and one on event weighting. It was shown that estimators just using event counts do not use the full information contained in the data. This is reflected in the fact that the figure of merit is smaller than in methods where events are weighted with an appropriate weight. The optimal weight for azimuthal asymmetries is $\cos (\varphi )$. It can also be shown that using this weight, the FOM is the same as in a maximum likelihood method reaching the Cramér–Rao limit of the lowest possible statistical error.

Among the estimators using event weights the method introduced in this paper has the advantage that no knowledge about the acceptance is required and no correction due to possible difference in luminosity has to be applied. On the contrary, the method even provides information on the azimuthal dependence of the acceptance. The method is easily extendable to more observables.

## Data Availability

This manuscript has no associated data or the data will not be deposited. [Authors’ comment: We don’t have associated data.]

## Covariance matrix of observables

The covariance matrix for the observables

$$\begin{aligned} \mathbf {y}_{\mathrm {obs}}= & {} \left( N^\uparrow , \sum _{\uparrow } \cos (\varphi _i),\right. \\&\left. \sum _{\uparrow } \cos ^2(\varphi _i), N^\downarrow , \sum _{\downarrow } \cos (\varphi _i),\sum _{\downarrow } \cos ^2(\varphi _i) \right) \end{aligned}$$

is

$$\begin{aligned} C= & {} \left( \begin{array}{cc} C_{\uparrow } &{} 0 \\ 0 &{} C_{\downarrow } \end{array} \right) \quad \, \quad \text{ with }\\ C_{\uparrow (\downarrow )}= & {} \left( \begin{array}{ccc} N^{\uparrow (\downarrow )} &{} \sum _{\uparrow (\downarrow )} \cos (\varphi _i) &{}\sum _{\uparrow (\downarrow )} \cos ^2(\varphi _i)\\ \sum _{\uparrow (\downarrow )} \cos (\varphi _i) &{} \sum _{\uparrow (\downarrow )} \cos ^2(\varphi _i) &{}\sum _{\uparrow (\downarrow )} \cos ^3(\varphi _i)\\ \sum _{\uparrow (\downarrow )} \cos ^2(\varphi _i) &{} \sum _{\uparrow (\downarrow )} \cos ^3(\varphi _i) &{}\sum _{\uparrow (\downarrow )} \cos ^4(\varphi _i)\\ \end{array} \right) . \end{aligned}$$

A derivation of the correlation between sums over events for different weights used here can be found in Ref. [11] (Appendix A).

## Figure of merit for cross ratio counting and weighting/fitting method

### FOM of counting methods

Assuming $P^\uparrow = -P^\downarrow$ and $\left\langle \cos (\varphi ) \right\rangle _L = -\left\langle \cos (\varphi ) \right\rangle _R =:\left\langle \cos (\varphi ) \right\rangle$, Eq. (5) simplifies to

$$\begin{aligned} {\hat{\epsilon }} = \frac{1}{\left\langle \cos (\varphi ) \right\rangle } \, \frac{\sqrt{\delta }-1}{\sqrt{\delta }+1} . \end{aligned}$$

Applying standard error propagation, one finds

$$\begin{aligned} \sigma _{\epsilon } = \frac{\mathrm {d}\epsilon }{\mathrm {d}\delta } \, \sigma _{\delta } \end{aligned}$$

with

$$\begin{aligned} \frac{\mathrm {d}\epsilon }{\mathrm {d}\delta } = \frac{1}{\left\langle \cos (\varphi ) \right\rangle } \, \frac{1}{(1+\sqrt{\delta })^2 \sqrt{\delta }} \end{aligned}$$

and

$$\begin{aligned} \sigma _{\delta } = \sqrt{\frac{1}{N^\uparrow _L} + \frac{1}{N^\uparrow _R} + \frac{1}{N^\downarrow _L} + \frac{1}{N^\downarrow _R} } \quad \delta . \end{aligned}$$

Using

$$\begin{aligned} N^\uparrow _{L,R}= & {} \frac{N_{\mathrm {tot}}}{2} \frac{1 \pm \left\langle \cos (\varphi ) \right\rangle \epsilon }{2} , \quad \text{ and } \\ N^\downarrow _{R,L}= & {} \frac{N_{\mathrm {tot}}}{2} \frac{1 \pm \left\langle \cos (\varphi ) \right\rangle \epsilon }{2} , \end{aligned}$$

with $N_{\mathrm {tot}} = N^\uparrow _{L} + N^\uparrow _{R} + N^\downarrow _{L} + N^\downarrow _{R}$, one finds

$$\begin{aligned} \frac{ \sigma _{\delta }}{\delta } = \frac{4}{\sqrt{N_{\mathrm {tot}}}} \, \frac{1}{\sqrt{1-\left\langle \cos (\varphi ) \right\rangle ^2\epsilon ^2}} . \end{aligned}$$

Using

$$\begin{aligned} \sqrt{\delta } = \frac{1+\left\langle \cos (\varphi ) \right\rangle \epsilon }{1 - \left\langle \cos (\varphi ) \right\rangle \epsilon } \end{aligned}$$

we finally arrive at

24 $$\begin{aligned}&\sigma _{\epsilon } = \frac{1}{\left\langle \cos (\varphi ) \right\rangle } \, \frac{1}{(1+\sqrt{\delta })^2 \sqrt{\delta }} \, \frac{4}{\sqrt{N_{\mathrm {tot}}}} \, \frac{1}{\sqrt{1-\left\langle \cos (\varphi ) \right\rangle ^2\epsilon ^2}} \delta \end{aligned}$$

25 $$\begin{aligned}&= \frac{1}{\left\langle \cos (\varphi ) \right\rangle } \, \sqrt{\frac{1- \left\langle \cos (\varphi ) \right\rangle ^2 \epsilon ^2 }{N_{\mathrm {tot}}}} \, . \end{aligned}$$

The FOM is given by

$$\begin{aligned} \text{ FOM }_{\epsilon } = N_{\mathrm {tot}} \frac{\left\langle \cos (\varphi ) \right\rangle ^2}{1-\left\langle \cos (\varphi ) \right\rangle ^2 \epsilon ^2} \end{aligned}$$

which agrees with Eq. (7). For the estimators in Eq. (6) the FOM is obtained by a similar procedure.

### FOM of weighting methods

Defining the luminosity factor $\ell _0 = {\mathcal {L}} \sigma _0 a_0$, Eq. (14) can be linearized around $\ell ^\uparrow =\ell _0$, $\ell ^\downarrow =\ell _0$, $\epsilon =\epsilon _0$ for $a_1=a_2=a_3=0$. Resulting in a system of linear equations

$$\begin{aligned} y_{\mathrm {model}} = A x_{\mathrm {para}} + y_0 \end{aligned}$$

with

$$\begin{aligned} x_{\mathrm {para}}&{=}&(\varDelta \ell ^\uparrow , \varDelta \ell ^\downarrow ,\varDelta \epsilon )^T , \quad y_0 {=} \ell _0 \left( 1, \frac{1}{2} \epsilon _0, \frac{1}{2},1, \frac{1}{2} \epsilon _0, \frac{1}{2} \right) ^T \\ A= & {} \, \left( \begin{array}{ccc} 1 &{} 0 &{} 0 \\ \epsilon _0 c_2 &{} 0 &{} \ell _0 c_2 \\ c_2 &{} 0 &{} 0 \\ 0 &{} 1 &{} 0 \\ 0 &{} -\epsilon _0 c_2 &{} -\ell _0 c_2 \\ 0 &{} c_2 &{} 0 \\ \end{array} \right) \, \quad \text{ and } \, \\ C= & {} \ell _0 \, \left( \begin{array}{cccccc} 1 &{} c_2 {\epsilon _0} &{} c_2 &{} 0 &{} 0 &{} 0 \\ c_2 \epsilon _0 &{} c_2 &{} c_4 \epsilon _0 &{} 0 &{} 0 &{} 0 \\ c_2 &{} c_4 \epsilon _0 &{} c_4 &{} 0 &{} 0 &{} 0 \\ 0 &{} 0 &{} 0 &{} 1 &{} - c_2 \epsilon _0 &{} c_2 \\ 0 &{} 0 &{} 0 &{} -c_2 \epsilon _0 &{} c_2 &{} -c_4 \epsilon _0 \\ 0 &{} 0 &{} 0 &{} c_2 &{} -c_4\epsilon _0 &{} c_4 \\ \end{array} \right) , \end{aligned}$$

with $c_n = \left\langle \cos (\varphi )^n \right\rangle$. The covariance matrix *C* is the same as in Appendix A except that we used here the expectation values instead of sum over events to arrive at an analytic expression. The covariance matrix for the parameters $(\varDelta \ell ^\uparrow , \varDelta \ell ^\downarrow ,\varDelta \epsilon )$, which is identical to the covariance matrix for the parameters $(\ell ^\uparrow , \ell ^\downarrow , \epsilon )$ since they just differ by a constant vector, is given by:

$$\begin{aligned} C_{\mathrm {para}} = (A^T \, C^{-1} \, A)^{-1} = \left( \begin{array}{ccc} \ell _0 &{} 0 &{} 0 \\ 0 &{} \ell _0 &{} 0 \\ 0 &{} 0 &{} \frac{c_2 - c_4 \epsilon _0^2}{2 c_2^2 \ell _0} \\ \end{array} \right) . \end{aligned}$$

For the FOM of $\epsilon _0$, replacing $\ell _0$ by $N_{\mathrm tot}/2$, one thus finds

$$\begin{aligned} \text{ FOM }_\epsilon = N_{\mathrm {tot}} \frac{\left\langle \cos ^2(\varphi ) \right\rangle ^2}{\left\langle \cos ^2(\varphi ) \right\rangle - \left\langle \cos ^4(\varphi ) \right\rangle \epsilon _0^2} , \end{aligned}$$

which agrees with Eq. (15).
